# The evaluation of sources of knowledge underlying different conceptual categories

**DOI:** 10.3389/fnhum.2013.00040

**Published:** 2013-02-21

**Authors:** Guido Gainotti, Pietro Spinelli, Eugenia Scaricamazza, Camillo Marra

**Affiliations:** ^1^Department of Neurosciences of the Policlinico Gemelli, Center for Neuropsychological Research, Catholic University of RomeRome, Italy; ^2^Department of Clinical and Behavioral Neurology, IRCCS Fondazione Santa LuciaRome, Italy

**Keywords:** interactions among sources of knowledge, animals vs. plant-life vs. artifact categories, sensory-motor model of semantic knowledge, visual-related knowledge, action-related conceptual knowledge

## Abstract

According to the “embodied cognition” theory and the “sensory-motor model of semantic knowledge”: (a) concepts are represented in the brain in the same format in which they are constructed by the sensory-motor system and (b) various conceptual categories differ according to the weight of different kinds of information in their representation. In this study, we tried to check the second assumption by asking normal elderly subjects to subjectively evaluate the role of various perceptual, motor and language-mediated sources of knowledge in the construction of different semantic categories. Our first aim was to rate the influence of different sources of knowledge in the representation of animals, plant life and artifact categories, rather than in living and non-living beings, as many previous studies on this subject have done. We also tried to check the influence of age and stimulus modality on these evaluations of the “sources of knowledge” underlying different conceptual categories. The influence of age was checked by comparing results obtained in our group of elderly subjects with those obtained in a previous study, conducted with a similar methodology on a sample of young students. And the influence of stimulus modality was assessed by presenting the stimuli in the verbal modality to 50 subjects and in the pictorial modality to 50 other subjects. The distinction between “animals” and “plant life” in the “living” categories was confirmed by analyzing their prevalent sources of knowledge and by a cluster analysis, which allowed us to distinguish “plant life” items from animals. Furthermore, results of the study showed: (a) that our subjects considered the visual modality as the main source of knowledge for all categories taken into account; and (b) that in biological categories the next most important source of information was represented by other perceptual modalities, whereas in artifacts it was represented by the actions performed with them. Finally, age and stimulus modality did not significantly influence judgment of relevance of the sources of knowledge involved in the construction of different conceptual categories.

## Introduction

Our knowledge of the world is mediated by two sorts of activities: (1) perceptual activities, which allow us to obtain information about external objects; (2) conceptual activities, which allow us to have internal representations of categories of objects. Even if both kinds of activities are necessary to understand the world we live in, the relationship between them is controversial and the amount of information that we have about their neuroanatomical and functional organization is very different. For example, we have detailed knowledge about the anatomical and functional organization of visual, auditory and somato-sensory systems, but the relationships between perceptual and conceptual activities are more controversial and there are strong debates about the format, the functional architecture and the neuroanatomical substrates of concepts.

Two main models have been advanced on this subject. According to the first model, proposed by Fodor (1975) and Pylyshyn (1973) and developed by Caramazza et al. (1990) and Patterson and Hodges (2000), concepts are represented in the brain in a formal, abstract manner, totally unrelated to the sensory-motor functions of the brain. According to this line of thought, modality-specific perceptual systems process sensory information up to a critical level (the structural description), that permits access to a unitary, abstract and amodal semantic system, where no trace of the previous sensory-motor mechanisms persists. On the contrary, according to the second model, proposed by Allport (1985) and Jackendoff (1987) and developed by Damasio (1989, 1990), Gainotti (1990, 2006), Saffran and Schwartz (1994), Gainotti et al. (1995), Martin (1998, 2007), Pulvermuller (1999), Martin and Chao (2001), Barsalou (2008), Kalénine et al. (2010), and Kiefer and Pulvermüller (2012) concepts are represented in the same format in which they were constructed by the sensory-motor system and can be considered as activity patterns distributed across different perceptual and motor attribute domains and integrated with language-mediated encyclopedic information.

In agreement with this second line of thought, anatomo-clinical studies of category-specific semantic disorders, which were prompted by a series of seminal papers by Warrington and coworkers (Warrington, 1975, 1981; Warrington and McCarthy, 1983, 1987; Warrington and Shallice, 1984) have suggested: (a) that different brain lesions may disrupt different categories of knowledge and (b) that the brain's organization of categorical knowledge may reflect the importance of the sensory-motor mechanisms which have mainly contributed to the development of different categories (the “differential weighting hypothesis”). In the original formulation of this general model, Warrington and Shallice (1984) contrasted the impairment of living beings with that of artifacts, and interpreted this dissociation as the consequence of the major role played by visual features in the identification of living things, and by functional features in the identification of nonliving (artifacts) categories.

### The sensory-functional theory and the embodied cognition/sensory-motor model of semantic representation

This interpretation, usually called the “sensory-functional theory”/SFT (Caramazza and Shelton, 1998; Tyler et al., 2000; Capitani et al., 2003; Ventura et al., 2005) has been unable to explain the complexity of the clinical data, because “functional features” are an heterogeneous class that includes actions accomplished with objects, notions about the objects' use and verbally-mediated encyclopedic knowledge. This fact was stressed by Buxbaum et al. (2000), Buxbaum and Saffran (2002), and Boronat et al. (2005). Within functional knowledge, these authors distinguished the function of an object from its manipulation and suggested that “manipulation” (which is related to a sensorimotor activity), might be the component most tightly linked to the “differential weighting” hypothesis. The same authors also showed that not only the properties denoted by the term “functional,” but also those subsumed by the term “sensory” are heterogeneous, because different types of sensory data could have a different weight in different kinds of semantic categories. Thus, the visual perception might play a leading role in the mental representation of animals and the somatosensory data in that of tools. These considerations led several authors (e.g., Saffran and Schwartz, 1994; Gainotti et al., 1995; Chao et al., 1999; Gainotti, 2000, 2006; Martin et al., 2000; Martin and Chao, 2001; Martin, 2007) to replace the “sensory-functional theory” with the “embodied cognition” theory (Barsalou, 2008) or with the “sensory-motor model of semantic knowledge,” (Gainotti, 2000, 2006; Martin and Chao, 2001; Martin, 2007), which takes into account various kinds of perceptual, functional, motor and verbally-coded properties, that can contribute to the construction of a conceptual representation.

### Studies which have tried to assess the weight of various kinds of information in the representation of different conceptual categories

Several studies have tried to assess the weight of various kinds of information in the representation of different conceptual categories, by asking normal subjects to make a subjective evaluation of the role of various perceptual, motor and language-mediated sources of knowledge in the construction of different semantic categories. After early studies that used dictionary definitions to obtain this information (Farah and McClelland, 1991; Caramazza and Shelton, 1998), two more recent sources of evidence have been used to evaluate the insight of normal subjects into the structure of concepts: (a) feature-listing tasks and (b) Likert-like scales devised to evaluate the weight of different “sources of knowledge” (Gainotti et al., 2009). In the feature-listing tasks, subjects are provided with a set of words and asked to generate features they associate with each word (Devlin et al., 1998; Tyler et al., 2000; Garrard et al., 2001; McRae and Cree, 2002; Vinson and Vigliocco, 2002; Cree and McRae, 2003; Vanoverberghe and Storms, 2003; Vinson et al., 2003; Vigliocco et al., 2004; McRae et al., 2005; Ventura et al., 2005; Zannino et al., 2006). In studies using Likert like scales to evaluate the weight of different “sources of knowledge,” verbal or pictorial stimuli which belong to different semantic categories, are shown to normal subjects. Using a graduated scale, the latter have to evaluate the relevance of different perceptual (visual, auditory, tactual, olfactory, and gustative), motor or language mediated information in the mental representation of the corresponding stimuli (Tranel et al., 1997; Gainotti et al., 2009; Carota et al., 2012; Hoffman and Lambon Ralph, 2013).

When a fine-grained taxonomy that distinguishes between different sensory modalities was used and the “performed action” (instead of the “function” category) was chosen, (e.g., Tranel et al., 1997; Vigliocco et al., 2004; McRae et al., 2005; Gainotti et al., 2009; Carota et al., 2012; Hoffman and Lambon Ralph, 2013) some results were consistently found with both methodologies used to evaluate the weight of different “sources of knowledge.” Thus, when the scores assigned to each modality were compared, visual information was consistently evaluated as the most important source of knowledge across all conceptual categories (Tranel et al., 1997; Cree and McRae, 2003; McRae et al., 2005; Gainotti et al., 2009; Carota et al., 2012; Hoffman and Lambon Ralph, 2013) and similar categorical clusters were obtained on the basis of the subjective ratings.

On the other hand, Hoffman and Lambon Ralph (2013) made a direct comparison between the verbal feature-listing task and a separate rating of each sensory-motor modality, and found that the former gave a distorted picture, with a strong bias toward visual form and function knowledge, whereas the latter uncovered a richer multimodal store of information, leading to novel insights about the structure of semantic space. The same authors also maintained that different results could be obtained by separately evaluating various “sources of knowledge” if the stimuli were presented as pictures or as written words.

### Aims of the present investigation

We set out to more thoroughly evaluate whether the subjective judgments of normal adults about the weight of different sources of knowledge in the development of different conceptual categories are consistent with the assumptions of the Embodied Cognition/sensory-motor model. To carry this out, we took into account a number of variables that have not yet been considered, or have given conflicting results. More specifically, we evaluated two main and two secondary issues:

1. The first main issue consisted of rating the influence of different sources of knowledge in the representation of animals, plant life and artifact categories, rather than in those of living and non-living beings, because many studies have already investigated this topic. The distinction (within the “living things”) between “animals” and “plant life” categories is suggested by two main facts: (a) these two categories can be dissociated by brain damage (see Caramazza and Shelton, 1998; Gainotti, 2005, 2010, 2011; Laiacona et al., 2006; Capitani et al., 2009 for reviews and discussions); (b) when hierarchical cluster analyses were used in feature listing studies (e.g., Cree and McRae, 2003) or in studies based on a separate rating of each sensory-motor modality (e.g., Gainotti et al., 2009; Hoffman and Lambon Ralph, 2013), a tripartite organization of knowledge was found, with three major clusters distinguishing between animals, plant life, and artifacts.
2. The second important aim of our study was to try to obtain a better understanding of the contribution deriving to the construction of various conceptual categories not from a single dominant source of knowledge, but from the association between several important sources of information. In a previous study (Gainotti et al., 2009) we showed that, if the visual data were considered as the most important source of knowledge for all categories considered, the next most relevant fonts of information were different for the living and the artifact categories. They consisted of other perceptual data in the case of the biological entities and of bodily-related actions and somato-sensory information in the case of artifacts. This method, was recently validated by Carota et al. (2012) in a two-stages fMRI study. In the first (behavioral) study, the body parts primarily and secondarily involved in actions accomplished with different categories of objects were evaluated by normal subjects using a Likert like scale. In the second (experimental) study the parts of the motor cortices activated by presentation of the corresponding words were investigated. This study showed that the hierarchy of bodily actions identified in the behavioral stage corresponded with the hierarchy of activation of the corresponding parts of the motor cortex in the fMRI study. Therefore, a more detailed study of these different patterns was necessary.
3. The third (less important) issue concerned the influence of the verbal or pictorial nature of the stimuli on the evaluation of the role played by various perceptual, motor, and language-mediated “sources of knowledge” in the construction of different conceptual categories.
4. The last (also less important) aim was to study these problems in a sample of elderly subjects who were unselected with respect to their educational level and working activities, because all previous studies based on feature-listing and the Likert-like scale methodology were conducted in young undergraduate students. On one hand, as humans tend to change their interests and their preferential approach to different aspects of experience during the lifespan, and as the sensory/motor theory is focused on experience as the basis of conceptual knowledge (Kellenbach et al., 2003; Noppeney et al., 2006; Connolly et al., 2007), it could be predicted that some differences would be found in the results obtained in a group of older and less educated subjects and those obtained by Gainotti et al. (2009) in a previous study carried out using the same material with young undergraduate students. For instance, due to the recent massive development of the media (mainly based on visual data), it is possible that the acquisition of knowledge about wild animals had been rated as relying above all on verbal material in elderly patients, but was based much more upon visual material in young students. It is also possible that for some categories of artifacts or fruits and vegetable (whose knowledge is partly based upon manipulation and action schemata) the relevance of action-related sources of knowledge, which are linked to the utilization of these schemata, might increase with age. The relationships among categories of knowledge might also change as a function of personal experience. Thus, in elderly subjects who, in their youth, might have used animals (e.g., horses or donkeys) as a means of transportation, the categories of animals and vehicles might be more closely linked than in young students who have never had this kind of experience.

On the other hand, if results similar to those obtained in young undergraduate students were found also in older, less educated subjects, this would contribute to confirming and expanding the significance of results obtained in previous investigations on this topic with respect to the sensory-motor model of semantic knowledge.

## Experimental procedure

### Subjects and materials

#### Material

Data were collected using two booklets that had a standardized format. Both contained 49 sheets of paper, each of which had a different item heading. Each items was represented in one booklet with the picture of the target stimulus and in the other booklet with the corresponding written word printed in big capital letters. The items consisted of 12 animals (6 pets and 6 wild animals), 16 plant life items (6 flowers, 5 vegetables, and 5 fruits) and 21 artifacts (5 pieces of furniture, 5 vehicles, 5 articles of clothing, and 6 tools). These items were as those used in our previous investigations (Gainotti et al., 2009). They were selected from a corpus of 100 colored pictures, on the basis of results obtained in a pilot study, conducted on an independent sample of 10 undergraduate students. The latter were requested to indicate the most familiar and prototypical items for each category and to judge each one for its imageability, concreteness, familiarity, and age of acquisition. Full agreement among the raters with respect to the high prototypicality of the stimuli was the main criterion used to select the most appropriate members of each category A second criterion was a good comparability between the different categories as for values concerning Age of Acquisition, Concreteness, Familiarity, and Imageability. No effort was made to have the same number of stimuli in each category. Subjects were requested to evaluate their familiarity with each stimulus object and the relevance of a number of sensory-motor sources of knowledge [visual shape and color, auditory, tactile, olfactory and taste (gustatory) perceptions and motor activities and language-mediated encyclopedic information] in constructing their knowledge of it. In a preliminary pilot study, we had also included within the visual sources of knowledge the observed movement of objects, because this aspect of vision, which is represented in the middle and superior temporal gyri (Beauchamp et al., 2002) was considered separately in studies conducted on undergraduate students by Cree and McRae (2003) and by Hoffman and Lambon Ralph (2013). This variable has been, however, excluded from the final version of our study because our elderly subjects, who were unselected with respect to their educational level and working activities, tended to confound the actions made on objects with their intrinsic movements.

In the present study, subjects had to assign a score ranging from 0 to 7 (0 denoted “no familiarity” and “no relevance” and 7, “very high familiarity” and “very high relevance”) to indicate the familiarity with each stimulus and to evaluate the relevance of each “source of knowledge” in constructing its representation. Five practice sheets, were given, which included items drawn from five different categories, saying, for instance, that “banana” is probably very familiar, because it is a common fruit, and that its knowledge could be mostly due to its visual properties (typical shape and yellow color), taste, and the actions made to peel it, whereas verbal descriptions (dictionary definitions) were probably less important and auditory sensations quite irrelevant. No subject had difficulty understanding the task, which entailed indicating the scores corresponding to the relevance of each “source” of knowledge on the response sheets.

#### Participants

The study was conducted on 100 normal adults (50 males and 50 females) of age ranging between 60 and 84 years of age and with an educational level ranging from 5 to 13 years of schooling. These subjects were recruited among the caregivers of patients attending the Neuropsychology Centre of the Policlinico Gemelli or in Centers for independent old people of the City of Rome who fulfilled the following criteria: (a) absence of neurological, medical, or psychiatric disorders that could influence their cognitive functions; (b) absence of severe visual and auditory disorders; (c) age and education corrected Mini Mental State Examination (MMSE) scores within the normal range.

To rule out the influence of response bias or of poor motivation during the task, we also excluded from analysis (d) subjects whose scores fell in the upper (from 4 to 7) or in the lower part (from 0 to 3) of the evaluation scale in more than 80% of the stimuli; (e) subjects who gave “odd” scores in more than 3 “sentinel items” (i.e., high scores on the “taste” modality for vehicles, on the “auditory” modality for fruits and on the “olfactory” modality for tools). Five subjects were excluded from analysis for reason (d) and 3 for reason (e). Therefore, the final number of participants was 92. Half of the subjects (mean age: 66.55 ± 6.01; educational level 9.7 ± 2.2; MMSE 28.7 ± 1.9) rated the relevance of each “source of knowledge” with stimuli presented in the written modality, and the other half (mean age: 66.44 ± 5.82; educational level 10.3 ± 2.8; MMSE 28.9 ± 1.3) made the same evaluation with stimuli presented in the pictorial modality. No significant differences were found for age, educational level and corrected MMSE scores, between subjects who had rated the relevance of each “source of knowledge,” with stimuli presented in the written and in the pictorial modality.

## Results

### Overall judgments of familiarity across the broad domains of animals, plant life, and artifacts

The first step of our analysis consisted of computing the mean values of familiarity across the broad domains of animals, plant life and artifacts, with and without considering separately the judgments given with verbal and pictorial stimuli. Table 1 summarizes data considered in this analysis.

**Table 1:** **Mean familiarity values and Standard Deviations (in brackets) for the domains of plant life, animals and artifacts: general scores and separate scores for presentation modality**.

	**Plant life (items = 32)**		**Animals (items = 24)**		**Artifacts (items = 42)**	
Familiarity	5.8 (0.60)		4.3 (1.02)		5.7 (0.89)	
	Pictorial	Verbal	Pictorial	Verbal	Pictorial	Verbal
	5.77 (0.60)	5.87 (0.61)	4.02 (1.08)	4.58 (0.90)	5.63 (0.99)	5.92 (0.76)

Data reported in Table 1 show that our subjects gave a different familiarity score to the different domains considered in our study; for example, animals were rated as significantly less familiar than plant life (*p* < 0.001) and artifacts (*p* < 0.001). In order to asses if judgments of familiarity could have been influenced by the verbal or pictorial nature of the stimuli administered, we carried out a two way ANOVA considering “Categories” and “Presentation Modality” as independent variables and scores on the judgment of familiarity as dependent variable.

The general analysis showed a significant general effect of the factor “Categories” [*F*_(2, 192)_, 57.85; *p* < 0.0001] suggesting a significant different general weight of the familiarity in the different categories. A significant general effect was also observed for the factor “presentation modality” [*F*_(1, 192)_; 5.16; *p* < 0.02] indicating a significant general effect of modality of presentation (i.e., by name or by picture) on the judgment of familiarity which resulted higher for the verbal than the pictorial presentation modality. No interaction was observed, however, between the two independent variables [*F*_(2, 192)_:1.06; *p* < NS] indicating that the presentation modality did not affect the judgment of familiarity across the different categories considered.

### Judgments of relevance of the various “sources of knowledge” across the domains of animals, plant life, and artifacts, considering the influence of the verbal or pictorial nature of the stimuli on these evaluations

Our next step was to assess whether the judgments of relevance of the various “sources of knowledge” were different across the broad domains of animals, plant life and artifacts and to consider the influence of the verbal or pictorial nature of the stimuli on these evaluations. Table 2 summarizes the data relevant to this analysis.

**Table 2:** **Mean values and Standard Deviations (in brackets) of the judgments of relevance of the various “sources of knowledge” in the domains of plant life, animals, and artifacts**.

	**Plant life (items n:32)**		**Animals (items n:24)**		**Artifacts (items n:42)**		**Picture**	**Verbal**
	**Picture**	**Verbal**	**Picture**	**Verbal**	**Picture**	**Verbal**	**F values**	**F values**
Visual “Shape”	**5.89**	**5.60**	**5.43**	**5.53**	**5.76**	**5.64**	8.9	0.49
	(0.36)	(0.51)	(0.47)	(0.41)	(0.40)	(0.38)	*p* < 0.001	*p*: ns
Visual “Color”	5.61	5.35	4.41	4.48	*3.96*	*3.73*	77.8	78.1
	(0.44)	(0.49)	(0.54)	(0.47)	(0.68)	(0.63)	*p* < 0.0001	*p* < 0.0001
Auditory	0.33	0.32	*2.71*	*2.69*	1.29	1.39	36.0	28.6
	(0.21)	(0.17)	(1.25)	(1.31)	(1.26)	(1.45)	*p* < 0.0001	*p* < 0.0001
Olfactive	*4.11*	*4.01*	1.84	1.99	0.87	0.82	153.9	135.6
	(1.02)	(1.02)	(0.85)	(0.87)	(0.50)	(0.60)	*p* < 0.0001	*p* < 0.0001
Gustatory	3.84	3.84	1.24	1.20	0.27	0.22	36.2	38.9
	(2.79)	(2.72)	(1.72)	(1.69)	(0.17)	(0.14)	*p* < 0.0001	*p* < 0.0001
Tactile	3.71	3.53	1.89	1.86	3.05	3.14	21.3	21.3
	(0.76)	(0.63)	(1.13)	(1.14)	(1.15)	(1.10)	*p* < 0.0001	*p* < 0.0001
Language	3.64	3.85	*3.99*	*4.04*	*3.55*	*3.97*	4.7	0.98
	(0.49)	(0.56)	(0.60)	(0.49)	(0.67)	(0.52)	*p* < 0.01	*p*: ns
Action/motricity	*4.25*	*4.19*	1.94	2.13	4.74	5.06	57.8	70.1
	(0.95)	(0.91)	(1.09)	(1.12)	(1.06)	(0.92)	*p* < 0.0001	*p* < 0.0001

Scores are analyzed separately by presentation modality.

Note: Columns report the values of the judgments of relevance of the different sources of knowledge across categories: the highest significant values (p < 0.05 after Bonferroni's correction) are reported in bold, the second highest are underlined and the next (third highest) are reported in italics.

In order to assess if the relevance of the various “sources of knowledge” was different across the different categories of animals, plant life and artifacts and whether it was influenced by the verbal or pictorial nature of the stimuli, we carried out a mixed MANOVA, followed by specific effect single ANOVAs and by *post hoc* Tukey test comparisons, in which Presentation Modality' were between factor and “Categories” within factor independent variables and scores of the various sources of knowledge taken into account as dependent variables. As the weight of the different sources of knowledge in each of the various broad categories of “Plant Life,” “Artifacts,” and “Animals” (column analyses) were not normally distributed, in a second analysis we investigated the differences among sources of knowledge using the Wilcoxon Matched paired tests, carried out separately for each broad category and type of presentation. The general analysis showed a significant effect within factor for “categories” [Wilks' lambda_(16, 176)_: 024; *p* < 0.0001] suggesting that the different sources of knowledge are judged as having a different weight in the construction of the different categories. A significant effect was also observed for the factor “presentation modality” [Wilks' lambda_(8, 88)_: 52; *p* < 0.006] indicating that the modality of presentation (by name or by picture) can influence the scores assigned by the subjects to the various sources of knowledge. Specific effect analyses showed that each source of knowledge had a significantly different weight in all categories with the exception of *Visual “Shape”* and *Language* after verbal presentation, which did not differ across categories.

When we analyzed the weight of each source of knowledge in each broad category, we observed that the visual form was considered as the most informative font for all the broad domains taken into account, and that the second source was different in the living categories of animals and plant life and in artifacts, because in the former the second source consisted of another visual property (i.e., color) whereas in artifacts it was represented by the actions performed with (or on) them. The differences between animals and plant life emerged at a later step, because the next most relevant sources of information in animals consisted of auditory perceptions (the typical sounds) and language (encyclopedic knowledge), whereas in plant life they consisted of actions (e.g., peeling, cutting, and stirring) and by olfactory and gustatory perceptions.

As for the second factor (modality of presentation), specific effect analyses showed that its effect was significant (*p* < 0.002) only in the “language” source of knowledge, which was higher by name [3.95 (± 0.25)] than by pictorial [3.72 (± 0.19)] presentation of the various items. A non-significant trend was also observed for the visual form (*p* < 0.07) and color (*p* < 0.08) which were higher by name than by pictorial presentation. The modality of presentation did not affect the scores assigned to the other sources of knowledge.

Finally a non-significant interaction (*p* < 0.06) was observed among the two independent variables (categories and presentation modality) indicating that the presentation modality does not influence the weights assigned by the subjects to the sources of knowledge across the different categories considered.

### Detailed evaluation of familiarity and of relevance of various “sources of knowledge” in the construction of different conceptual categories

As results of the previous section showed that the modality of presentation has a significant effect only on the weight assigned to the source of knowledge “language,” we did not analyze separately the evaluations given with verbal and pictorial stimuli, when we tried to more analytically consider the judgments of familiarity and the relevance attributed to various sources of knowledge in the construction of more specific domains within the above mentioned general categories. Therefore, this evaluation was made by grouping together scores obtained for each source of knowledge with verbal and pictorial stimuli.

Data concerning this analysis are reported in Table 3.

**Table 3:** **Overall mean values of familiarity and judgments of relevance of the various sources of knowledge for specific semantic categories of animals, plant life, and artifacts**.

	**Familiarity**	**Language**	**Visual shape**	**Visual color**	**Action/motricity**	**Auditory**	**Tactile**	**Olfactive**	**Taste**
*Fruits*	**6.27**	3.89	5.97^**^	5.63^*^	*4.98*	0.27	**4.15**	**4.14**	6.09^**^
	0.33	0.54	0.37	0.48	0.44	0.21	0.50	0.95	0.38
*Vegetables*	5.93	3.46	5.71^**^	5.49^*^	4.58	0.33	*3.59*	*3.51*	5.75^**^
	0.34	0.48	0.38	0.39	0.66	0.16	0.58	1.09	0.56
*Flowers*	5.35	3.86	5.59^**^	5.34^**^	3.30	0.36	3.21	4.45^*^	0.38
	0.62	0.50	0.53	0.53	0.58	0.20	0.65	0.83	0.23
*Pets*	5.12	4.10^*^	5.69^**^	4.49^*^	2.98	**3.38**	2.83	2.54	*2.24*
	0.62	0.39	0.37	0.40	0.66	1.14	0.76	0.71	1.91
*Wild animal*	3.48	3.93^*^	5.28^**^	4.41^*^	1.10	*2.02*	0.92	1.30	0.21
	0.61	0.49	0.41	0.59	0.41	1.01	0.31	0.44	0.08
*Tools*	**6.16**	3.58	5.74^**^	3.32	5.36^*^	1.01	3.39	0.31	0.27
	0.60	0.59	0.31	0.44	0.60	0.62	0.74	0.17	0.20
*Furniture*	**6.17**	3.78	5.80^**^	3.95	5.20^*^	0.65	3.20	0.80	0.26
	0.59	0.50	0.51	0.64	0.74	0.36	0.99	0.47	0.17
*Clothes*	5.79	3.43	5.57^**^	4.32	4.82^*^	0.51	*3.78*	1.21	0.25
	0.72	0.53	0.30	0.54	0.90	0.36	0.93	0.46	0.13
*Vehicles*	4.92	4.30^*^	5.71^**^	3.90	4.10^*^	**3.27**	1.98	1.16	0.22
	1.02	0.57	0.42	0.58	1.25	1.43	1.04	0.51	0.12
*P* value	*p* < 0.0001	*p* < 0.0001	*p* < 0.0001	*p* < 0.0001	*p* < 0.0001	*p* < 0.0001	*p* < 0.0001	*p* < 0.0001	*p* < 0.0001

Note: Columns report the values of familiarity and of the different sources of knowledge across categories: the significantly (p < 0.05 after Bonferroni's correction) highest values obtained in familiarity and in specific sources of knowledge on each category are reported in bold (first values) or italics (second values). The weight of the various sources within each category are reported in rows. The highest significant values (p < 0.05 after Bonferroni's correction) obtained in each category on the various sources of knowledge are marked with asterisks [

** first value(s);

* second value(s)].

These data were analyzed with a general MANOVA, followed by specific effect single ANOVAs,, which considered the “categories” as independent variables and the “familiarity ratings” and the “sources of knowledge” as dependent variables. This general analysis was highly significant, (Wilks' lambda 0.0003 *p* < 0.0001) suggesting that the familiarity with the various categories was different and that various sources of knowledge play a different role in the construction of the categories considered in our study. Specific comparisons were carried out by means of single ANOVAs followed by *post hoc* Tukey tests for unequal sample size to analyze the single effects of each source of knowledge in the various categories (columns analyses). On the other hand, as the data in the categories were not normally distributed (rows analyses), Friedman's Matched Pairs Rank Order analyses of variance (Siegel, 1956) were carried out to compare the different weight of each source in each category.

The study of subjective familiarity has provided more precise details about results previously described in Table 2 by showing that fruits, tools and furniture are generally rated as the most familiar, whereas wild animals are by far rated as least familiar. The analysis of the sources of knowledge, on the other hand, shows that all the sources considered in our study have a very different influence on the organization of the specific categories taken into account. Data reported in the different columns of Table 3 confirm the predominant role attributed by our subjects to the visual modality, particularly to shape, as the main source of knowledge for almost all the categories considered in our study. The only exception was “taste,” which was rated as slightly more important than shape for fruits and vegetables; however, this difference was not significant. Shape and taste are, therefore ranked first ex aequo for this category. Color was also evaluated as very important for all the living categories (fruits, vegetables, flowers, and wild or domestic animals), whereas all other “perceptual” modalities were less relevant (or relevant only for specific categories). Thus, (as we have already noticed) taste is very relevant only for fruits and vegetables, and a very similar pattern is attributed to smell (which was rated high for flowers, fruits and vegetables and, to a lesser extent, for pets). On the other hand, the action-related sources of knowledge are considered as only slightly less relevant than the visual shape in the case of the tool and furniture categories, and are also very important in the representation of other artifacts and fruits and vegetables. This last finding is probably due to the fact that, just as in the case of artifacts, part of our knowledge of fruits and vegetables comes from actions (such as peeling, cutting, cooking, or eating) made on them. For the same reason, a pattern of relevance similar to that of the typical actions is observed when we consider the somato-sensory data, which are judged as important for the categories of fruits and vegetables, clothing, tools, and furniture. Apart from these clusters, a rather similar pattern is presented by language and auditory information. Language (encyclopedic knowledge) is considered as important, but not critical, for almost all the categories but particularly relevant for animals and vehicles. Similarly, the role of auditory information is judged as important only for pets and vehicles (probably because both make typical sounds).

### Comparisons of scores obtained by young and elderly subjects on the judgments of relevance of the various sources of knowledge in the construction of different semantic categories

Table 4 reports the rank order of the judgments of relevance of the various sources of knowledge in the construction of different semantic categories obtained in our previous study on young adults (Gainotti et al., 2009) and in the present investigation.

**Table 4:** **Rank order of the judgments of relevance of the various sources of knowledge for the specific semantic categories of animals, plant life and artifacts in Young (Y) and Elderly (E) subjects**.

		**Language**	**Visual**	**Action/motricity**	**Auditory**	**Tactile**	**Olfactive**	**Taste**
*Fruits*	*E*	6	1	3	7	4	4	1
	*Y*	6	1	4	7	4	3	1
*Vegetables*	*E*	6	1	3	7	4	4	1
	*Y*	6	1	5	7	4	3	2
*Flowers*	*E*	3	1	4	6	4	2	6
	*Y*	3	1	5	6	4	2	7
*Pets*	*E*	2	1	4	3	4	6	6
	*Y*	2	1	3	3	3	3	7
*Wild animal*	*E*	2	1	4	3	6	4	7
	*Y*	2	1	4	3	5	5	7
*Tools*	*E*	3	1	2	5	3	6	6
	*Y*	4	1	2	5	3	6	6
*Furniture*	*E*	3	1	2	5	4	5	7
	*Y*	4	1	2	5	3	6	6
*Clothes*	*E*	4	1	2	6	3	5	7
	*Y*	4	1	2	6	3	5	7
*Vehicles*	*E*	2	1	2	4	5	6	7
	*Y*	3	1	2	3	5	6	7

Note: The numbers represent the rank achieved by each category in the present study and in the previous study on young subjects by Gainotti et al. (2009).

It was impossible to make more analytical and statistical comparisons between the two sets of data, for two main reasons. First, the sources of knowledge taken into account in the two studies were not exactly the same. In particular, visual shape and color were rated separately in the present investigation, whereas in the previous study they had been pooled together under the heading of “visual sources of knowledge.” Second, in the first study subjects had been given a sheet with both picture and name of each stimulus, whereas in this second study pictures and names were presented separately to different populations. For this reason, in Table 4 we matched only the ranks of the ratings obtained in each modality by young and elderly patients and for the latter we made a mean of the ratings obtained after picture and name presentation for all single items.

The representation (Table 4) of the rank of relevance in young and elderly people for all the categories and all the sources of knowledge shows, in any case, that if changes can be found as a function of age in the importance of various source of knowledge for the conceptual representation, these changes are really minimal. The visual sources of knowledge were ranked first by both young and elderly subjects in almost all the semantic categories considered. Similarly, in both young and in elderly subjects, the second rank consisted of: (a) action based knowledge in almost all the artifact categories; and (b) language in both domestic and in wild animals. Furthermore, in both young and in elderly subjects the third rank consisted of: (a) the auditory information for wild animals and (b) the tactile sources of knowledge for almost all the artifact categories. The only slight difference between elderly and young subjects was in the “vegetable” category, in which action-related sources of knowledge were ranked higher (3rd) by elderly than young (5th) subjects. This slight difference may be due to the fact that the knowledge of vegetable is in part based on manipulation and action schemata, whose frequent utilization can increase with age.

### Cluster analysis on data obtained in the different conceptual categories considered in our study

**Figure 1:**
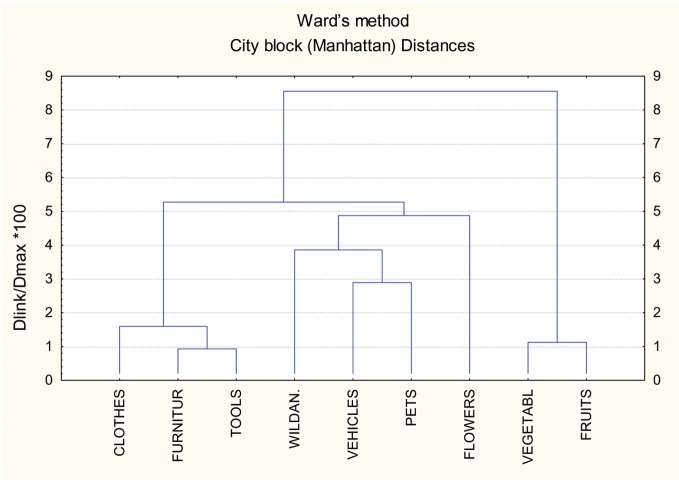
**Cluster analysis of the semantic categories grouped in terms of salience of the different sources of knowledge**.

In order to provide a representation of how our specific categories of animals, plant life, and artifacts group, in terms of relative importance of the different sources of knowledge, we conducted a cluster analysis, considering the distance among the various categories as an index of their similarity, in terms of weight of the various sources of knowledge. Furthermore, to maximize the distance among categories, rather than among sources of knowledge, a rather strict form of “cases tree clustering” (i.e., the “City Block ‘Manhattan’ Distance”) representing a complete linkage method, was used for the cluster analysis. The results are shown in Figure 1.

There are three major clusters in Figure 1, each corresponding basically, but not exactly, to the distinction between plant life, animals, and artifacts. On the extreme right of the dendrogram there is a cluster consisting of fruits and vegetables. This cluster is very close to the left side of a larger cluster, consisting of two subgroups, including flowers, pets, vehicles, and wild animals. The last, most left-sided cluster includes tools, furniture, and clothes. Thus, fruits and vegetables are located on the right side of the dendrogram and manipulable objects on the left side. In the center of the dendrogram is a large and heterogeneous group of categories, which includes on its right side flowers, then domestic and wild animals and vehicles. The association between pets and vehicles is probably due to the relevance that our subjects attributed to language and typical sounds, as important sources of knowledge of both these categories, as is shown by data reported in Table 3.

## Discussion

The goal of the present investigation was to more thoroughly analyze the problem of the relevance of different sources of knowledge in the construction of various conceptual categories, by taking into account a number of variables that have never been considered or have given conflicting results. Our expectation was that a more thorough knowledge of the relevance of different sources of knowledge in the construction of various conceptual categories would help choose among the different theories on the representation of concepts discussed in the section “Introduction,” and would confirm and extend results obtained in previous investigations (e.g., Tranel et al., 1997; Vigliocco et al., 2004; McRae et al., 2005; Gainotti et al., 2009; Carota et al., 2012; Hoffman and Lambon Ralph, 2013), thus providing further support for the “sensory-motor model of semantic knowledge,” (Gainotti, 2000, 2006; Martin and Chao, 2001; Martin, 2007; Kalénine et al., 2010).

With this aim in mind, we focused on broad semantic categories and rated stimulus familiarity and the influence of different sources of knowledge in the representation of animals, plant life, and artifact categories, rather than in living and non-living beings, which has been done in many previous studies on this topic.

The distinction between “animals” and “plant life” in the “living” categories was confirmed by results of the present investigation, because, even if visual features (shape and color) were considered as the most informative sources of knowledge for all the living categories, an important difference between animals and plant life emerged at a later step. In fruits and vegetables the next most relevant sources of information consisted of olfactory and gustatory perceptions and by actions (e.g., peeling, cutting, and stirring), whereas in animals they consisted of auditory perceptions (typical sounds) and language (encyclopedic knowledge).

A cluster analysis also allowed distinguishing fruits and vegetables from animals, because the former made a small distinct cluster, whereas the latter entered into a larger heterogeneous cluster, which was intermediate between “fruits and vegetables” on one side and “artifacts” on the other side. All these data are consistent with results obtained in previous studies, carried out with young undergraduate students by Tranel et al. (1997), Cree and McRae (2003), Vinson et al. (2003), Vigliocco et al. (2004), Gainotti et al. (2009), and Hoffman and Lambon Ralph (2013).

**Figure 2:**
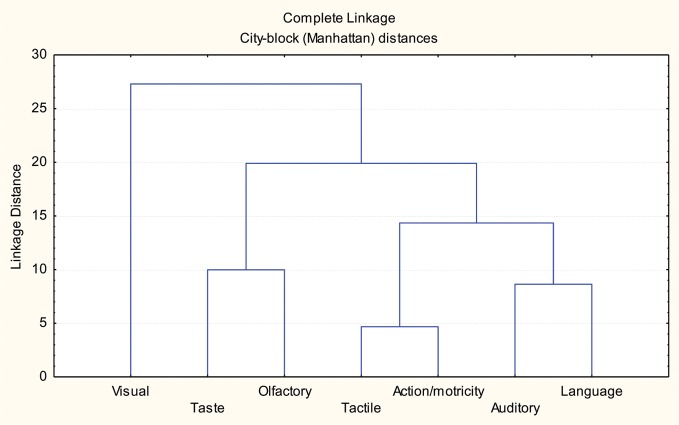
**Cluster analysis carried out to explore how the sources of knowledge group together in terms of their specific weight in characterizing the items of all the categories considered in the study**.

Also consistent with results of previous investigations and perfectly in line with the principles of the “sensory-motor model of semantic knowledge,” were the associations between sources of knowledge characteristic of living beings and artifacts, because, even though visual features were considered as the most informative sources of knowledge for all the biological and artifacts categories, the second most important font was different for the living categories and for artifacts. In all the biological categories the second most important source consisted of another perceptual property (that was different for the specific domains of animals and plant-life), whereas in artifacts it was represented by the actions performed with (or on) them and by the correlative somato-sensory information. An evidence supporting the claim of a strict connection between action schemata and the correlative somato-sensory information comes from a cluster analysis derived from the sources of knowledge instead than from the categories and reported in Figure 2.

In this case, the cluster shows a tight linkage between these two sources of knowledge. Furthermore, the same cluster analysis confirms: (a) that visual data stand apart from all the other sources of knowledge; (b) that a significant linkage exists between taste and smell (in fruits and vegetables) and between auditory data and language (in animals and vehicles).

The patterns of associations between visual and other perceptual information in living beings and between visual and action related information in tools and other artifacts have been reported in previous studies by Tranel et al. (1997), Vigliocco et al. (2004), Gainotti et al. (2009), and by Hoffman and Lambon Ralph (2013). These associations between visual and other perceptual modalities in the case of biological entities and between visual and action related information in the case of artifacts are consistent with the neuroanatomical models of these categories, drawn from the “sensory-motor model of semantic knowledge,” (Gainotti et al., 2009; Gainotti, 2010; Kalénine et al., 2010). These models, indeed, suggest that biological entities should be represented at the convergence between the ventral stream of visual processing and other perceptual inputs, whereas tools and other artifacts should be represented at the convergence between the dorsal stream of visual processing and the motor and somatosensory cortices. Surely, the anterior parts of the temporal lobes (where the ventral stream converges with auditory, olfactory and gustatory inputs), should play a critical role in the representation of biological entities (Gainotti, 2000, 2005, 2010), whereas the left frontoparietal, sensorimotor cortices (where the dorsal stream converges with body-related and action oriented structures), should play a major role in the representation of artifacts (Gainotti, 2000; Kellenbach et al., 2003; Martin, 2007; Kalénine et al., 2010). A second less important aim of our study was to evaluate the influence of the verbal or pictorial nature of the stimuli on the judgment of relevance of various perceptual, motor, and language-mediated “sources of knowledge” in the construction of different conceptual categories. We included this because in a previous study on this topic (Gainotti et al., 2009), each item had been represented with both a picture and a written word printed in big capital letters. This was criticized by Hoffman and Lambon Ralph (2013), who argued that a pictorial form could bias subjects toward giving higher ratings for visual sources of knowledge. Our results do not confirm this claim, because they have shown that the verbal or pictorial modality of presentation affects the familiarity feelings more than the judgment of relevance of the sources of knowledge. Stimuli were, indeed, judged as more familiar when they were presented in the verbal than in the pictorial modality, whereas the modality of presentation exerted a significant effect only on the weight assigned to the source of knowledge “language,” which was higher when the stimulus was presented by name than by picture. On the contrary, neither the verbal nor the pictorial modality of presentation affected the scores assigned to the other sources of knowledge and no interaction was observed between categories and presentation modality, indicating that the verbal or pictorial nature of the stimuli did not influence the weights assigned by the subjects to the sources of knowledge across the broad domains taken into account.

A third variable considered in our study was the influence of age (and of experience related factors), because we intended to check if results obtained in previous studies by young undergraduate students could be generalized to older adults, who were unselected as to their educational level and working activities. Our results showed that this generalization is possible, because only small differences were found between the judgments made by young undergraduate students and elderly subjects regarding the relevance of different sources of knowledge in the construction of various conceptual categories. Our elderly subjects showed, indeed, a systematic tendency to consider the visual modality as the main source of knowledge for all categories of animals, plant-life and artifacts and estimated that, after the visual information the second most important source is different for the living categories and artifacts. In the living categories the second most important source of knowledge was a perceptual property whereas in the case of artifacts it was represented by the actions performed with (or on) them. All these findings are consistent with data obtained with different methodologies in young undergraduate students by Tranel et al. (1997), Vigliocco et al. (2004), Gainotti et al. (2009), and Hoffman and Lambon Ralph (2013), confirming that data obtained in a sample of young students can be generalized to older subjects, unselected with respect to their educational level and working activities. The only differences between results of our previous study and of the present investigation that could suggest a (very mild) influence of age-related experience on the sources of knowledge underlying the different semantic categories concerned: (a) the greater relevance of the action-related sources of knowledge in elderly than in young subjects for the vegetables category; (b) the tighter association between animals and vehicles found in our dendrogram in comparison with that obtained in the study conducted on young subjects. We had, indeed, hypothesized in the introduction: (a) that for some categories of artifacts or fruits and vegetable, whose knowledge in partly based upon manipulation and action schemata (Gainotti, 2010) the relevance of action-related sources of knowledge could increase with age; (b) that the categories of animals and vehicles could be more closely linked in elderly patients, who in their young age had perhaps used some animals (e.g., horses or donkeys) as a mean of transport than in young students, who had never proved this kind of experience. The first point has been (at least in part) confirmed by our data, whereas the relevance of the second point remains problematic, because an association between animals and vehicles has recently been reported also in young people by Hoffman and Lambon Ralph (2013), who have noticed that the knowledge of both vehicles and animals is based on typical sounds and motion information. Furthermore, although many studies have treated artifacts as a single category, there are some cases of patients with non-living deficits who showed relatively preserved knowledge of vehicles. For instance, patient YOT (Warrington and McCarthy, 1987) showed poor comprehension of manipulable objects but more intact knowledge of large artifacts, many of which were vehicles, and similar results were observed in patients KE (Hillis et al., 1990) and GP (Cappa et al., 1998). It seems, therefore, safe to conclude that age-related factors, based on changes in the external milieu or on personal experiences have a very limited impact on judgments concerning the relevance of different sources of knowledge in the construction of various conceptual categories.

## Concluding remarks

In conclusion, results of the present research confirm: (a) that a distinction can be made between “animals” “plant life” and artifacts on the basis of their prevalent sources of knowledge; (b) that this distinction is not based on the prevalence of visual information in living beings and of functional information in artifact categories, but rather on the convergence of visual with other perceptual data in the biological categories and of visual with action and somatosensory data, in the representation of tools and other artifact categories; (c) that age and stimulus modality have a very limited influence on the weights assigned by normal subjects to the sources of knowledge underlying various kinds of biological and artifact categories. All these data confirm and extend results obtained in previous investigations, and provide further support for the “sensory-motor model of semantic knowledge.”

### Conflict of interest statement

The authors declare that the research was conducted in the absence of any commercial or financial relationships that could be construed as a potential conflict of interest.
